# Notochordal cell matrix as a bioactive lubricant for the osteoarthritic joint

**DOI:** 10.1038/s41598-018-27130-9

**Published:** 2018-06-11

**Authors:** S. A. H. de Vries, M. van Doeselaar, H. J. Kaper, P. K. Sharma, K. Ito

**Affiliations:** 1 Orthopaedic Biomechanics, Department of Biomedical Engineering, Eindhoven University of Technology, Eindhoven, The Netherlands; 2 Department of Biomedical Engineering, University of Groningen, University Medical Centre Groningen, Groningen, The Netherlands; 3 Department of Orthopaedics, University Medical Center Utrecht, Utrecht, The Netherlands

## Abstract

Notochordal cell derived matrix (NCM) can induce regenerative effects on nucleus pulposus cells and may exert such effects on chondrocytes as well. Furthermore, when dissolved at low concentrations, NCM forms a viscous fluid with potential lubricating properties. Therefore, this study tests the feasibility of the use of NCM as a regenerative lubricant for the osteoarthritic joint. Chondrocyte-seeded alginate beads were cultured in base medium (BM), BM with NCM (NCM), or BM with TGF-β1 (TGF), as well as BM and NCM treated with IL-1β. NCM increased GAG deposition and cell proliferation (stronger than TGF), and GAG/DNA ratio and hydroxyproline content (similar to TGF). These effects were maintained in the presence of IL-1β. Moreover, NCM mitigated expression of IL-1β-induced IL-6, IL-8, ADAMTS-5 and MMP-13. Reciprocating sliding friction tests of cartilage on glass were performed to test NCM’s lubricating properties relative to hyaluronic acid (HA), and showed a dose-dependent reduction in coefficient of friction with NCM, similar to HA. NCM has anabolic and anti-inflammatory effects on chondrocytes, as well as lubricating properties. Therefore, intra-articular NCM injection may have potential as a treatment to minimize pain while restoring the affected cartilage tissue in the osteoarthritic joint.

## Introduction

Articular cartilage (AC) is a layer of smooth hydrated tissue that covers the articulating surfaces of bones in fluid filled synovial joints. Together with the synovial fluid, it provides low friction in these joints during motion. Osteoarthritis (OA), a degenerative joint disease, affects the AC as well as the synovium and subchondral bone, leading to painful articulating dysfunction. Knee OA is one of the leading causes for pain and disability worldwide, with estimates suggesting 9.3 million affected people in the US alone^1^.

OA is initially treated conservatively, with exercise and pain medication. In later stages, non-steroidal anti-inflammatory drugs or intra-articular steroid injections are prescribed^2^. Another treatment option is viscosupplementation^3^ i.e. intra-articular injection of hyaluronic acid (HA), a large polysaccharide that is naturally found in synovial fluid^4^. HA increases the viscosity^5^ of the synovial fluid in addition to providing viscoelasticity thereby contributing to hydrodynamic lubrication of the joint^6,7^. Furthermore, due to molecular interactions at the cartilage surface, it contributes to boundary lubrication as well^8^. With OA, HA degrades resulting in a decreased concentration and low molecular weight fragments, which affects the lubricating properties of synovial fluid^9^. Injection of HA into the joint aims to increase synovial fluid viscosity and minimize pain to postpone total knee replacement. Although meta-analyses provide contradicting results regarding the efficacy of HA visco-supplementation^10^, it is generally considered as a safe and effective treatment for painful knee OA^11^. Despite HA’s positive effects, it only provides symptomatic relief and does not restore the affected cartilage to a healthy state. Therefore, other options should be explored.

In the field of intervertebral disc (IVD) regeneration, notochordal cells (NCs) have received considerable attention. They produce soluble factors capable of stimulating nucleus pulposus cell (NPC) matrix production and proliferation^12–15^ as well as differentiation of bone marrow-derived stem cells (BMSCs) to a chondrogenic phenotype^16,17^. An alternative to NC-secreted factors is the direct use of lyophilized and pulverized porcine NC matrix (NCM). This material was applied in an *in vitro* culture of bovine NPCs^18^, where it exerted an even stronger anabolic effect compared to NC-secreted soluble factors. Moreover, intradiscal injection of NCM in a canine *in vivo* study had anabolic and anti-inflammatory effects and increased IVD hydration^19^. Since NPCs closely resemble articular chondrocytes, NCM may also have the potential to stimulate these cells. Moreover, when dissolved at a low concentration in aqueous media, NCM forms a viscous fluid that may have lubricating properties, similar to HA.

Since human NCM can only be obtained from fetal and young donors, it’s use faces ethical and practical issues. Porcine NCM may however be a viable alternative as it readily available. As a xenogenic source, the use of porcine derived tissue products would not be novel. Porcine tissue derived products, such as ligaments^20^ and decellularized heart valves^21^ have already been clinically applied. Moreover, a study testing the regenerative potential of NC-conditioned medium (NCCM) from several species on human NPCs, found even stronger effects of porcine NCCM than human NCCM, suggesting a strong translational potential^13^.

The aim of the current study was to investigate whether it is feasible to use porcine NCM as a biomaterial with lubricating properties, that could simultaneously stimulate chondrocytes to restore the affected cartilage within the OA joint. First, the regenerative potential of porcine NCM on bovine chondrocytes was investigated in an *in vitro* alginate bead culture. Second, it was investigated whether NCM could also stimulate chondrocytes in the presence of an inflammatory stimulus. Lastly, reciprocating sliding cartilage on glass friction tests were performed to test NCM’s lubricating properties relative to and in combination with hyaluronic acid (HA).

## Results

### NCM’s regenerative potential

**Figure 1:**
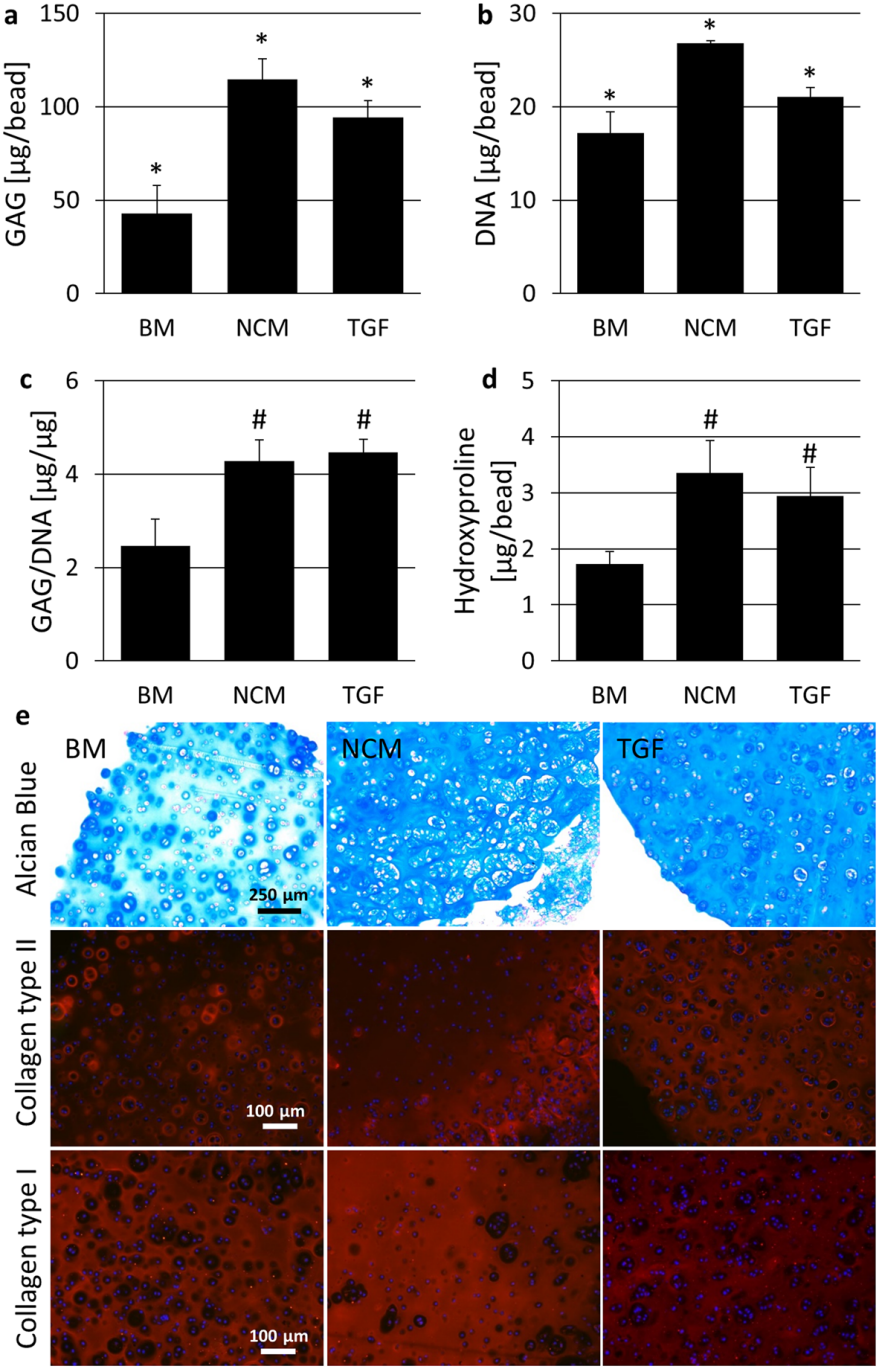
Porcine NC-rich NP matrix (NCM) induced an anabolic response of bovine chondrocytes (**a**) Glycosaminoglycan (GAG) and (**b**) DNA content per alginate bead seeded with bovine chondrocytes, (**c**) GAG per DNA and (**d**) hydroxyproline content per bead. Values represent means + standard deviations, n = 5 per group. *Indicates p < 0.05 compared to all other groups, ^#^indicates p < 0.05 compared to base medium (BM). (**e**) Alcian blue staining confirms increased GAG deposition with NCM and BM supplemented with 10 ng/ml TGF-β1 (TGF) compared to BM. Collagen immunohistochemistry shows increased collagen type II at the edge of the bead and more diffuse collagen type II deposition with TGF. Collagen type I staining intensity appears to be increased with NCM.

Both addition of NCM and TGF resulted in increased GAG content compared to BM (Fig. 1a). Furthermore, GAG content with NCM was significantly higher compared to TGF. A similar pattern is observed with DNA content, which increased with TGF compared to BM, but increased further with NCM (Fig. 1b). These data lead to a similar increased GAG per DNA ratio for NCM and TGF compared to BM (Fig. 1c). Also, hydroxyproline, as a measure for collagen content, increased with both NCM and TGF relative to BM (Fig. 1d). Alcian blue staining confirmed the increased GAG content with NCM and TGF (Fig. 1e). From collagen immunostainings, collagen type II deposition appeared to be stimulated with NCM mainly at the edge of the bead, but especially with TGF compared to BM. Collagen type I deposition appeared not to be affected by TGF, whereas beads cultured in NCM stained more intense.

**Figure 2:**
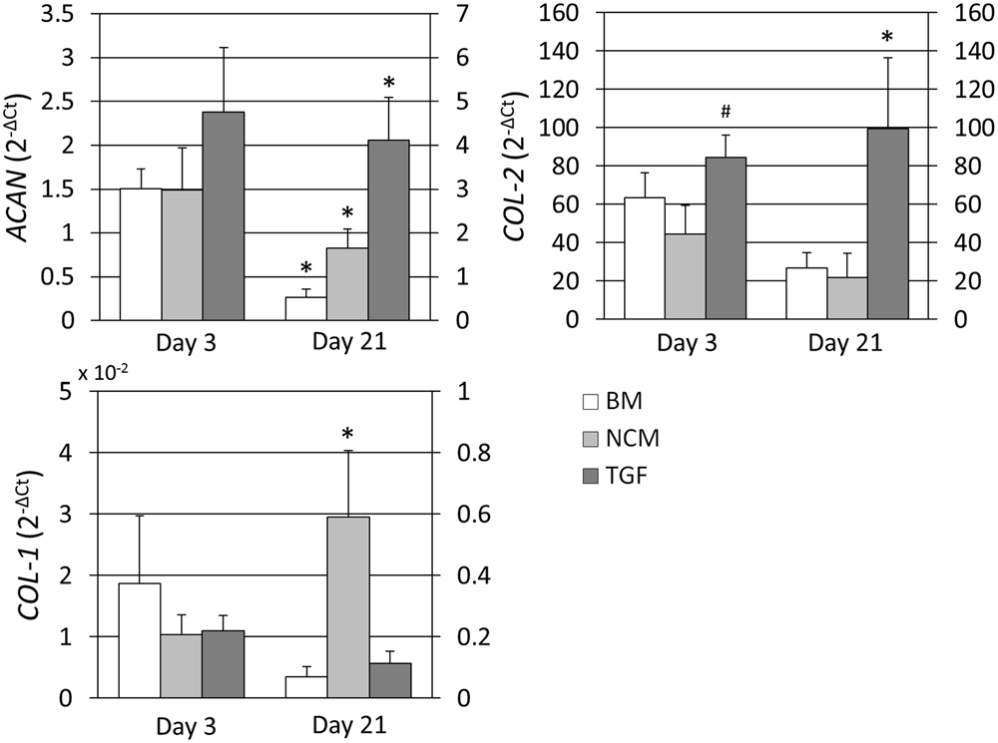
NC-rich nucleus pulposus matrix (NCM) has distinct anabolic effects in chondrocyte-seeded alginate beads. *ACAN*: aggrecan; *COL*-2: collagen type II alpha 1; *COL*-1: collagen type I alpha 1. Expression levels are relative to 60 S ribosomal protein L13 (*RPL13*). Values are means + standard deviations, n = 5 independent repeats per group. *Indicates p < 0.05 compared to all other groups at the same time point, ^#^indicates p < 0.05 compared to base medium (BM).

To determine the anabolic effect of NCM at the gene level, gene expression analysis of *ACAN*, *COL*-2 and *COL*-1 was performed (Fig. 2). At day 3 no differences in *ACAN* expression was observed between culture groups. At day 21 however, expression of ACAN increased with both NCM and TGF compared to BM, and with TGF compared to NCM. Expression of *COL*-2 was not significantly different with NCM compared to BM at day 3 and 21, but was significantly higher with TGF compared to NCM at day 3, and compared to BM and NCM at day 21. At day 3 no significant differences in *COL*-1 expression were observed between culture groups, however *COL*-1 expression was significantly higher in NCM compared to BM and TGF at day 21.

### NCM’s potential in an inflammatory environment

**Figure 3:**
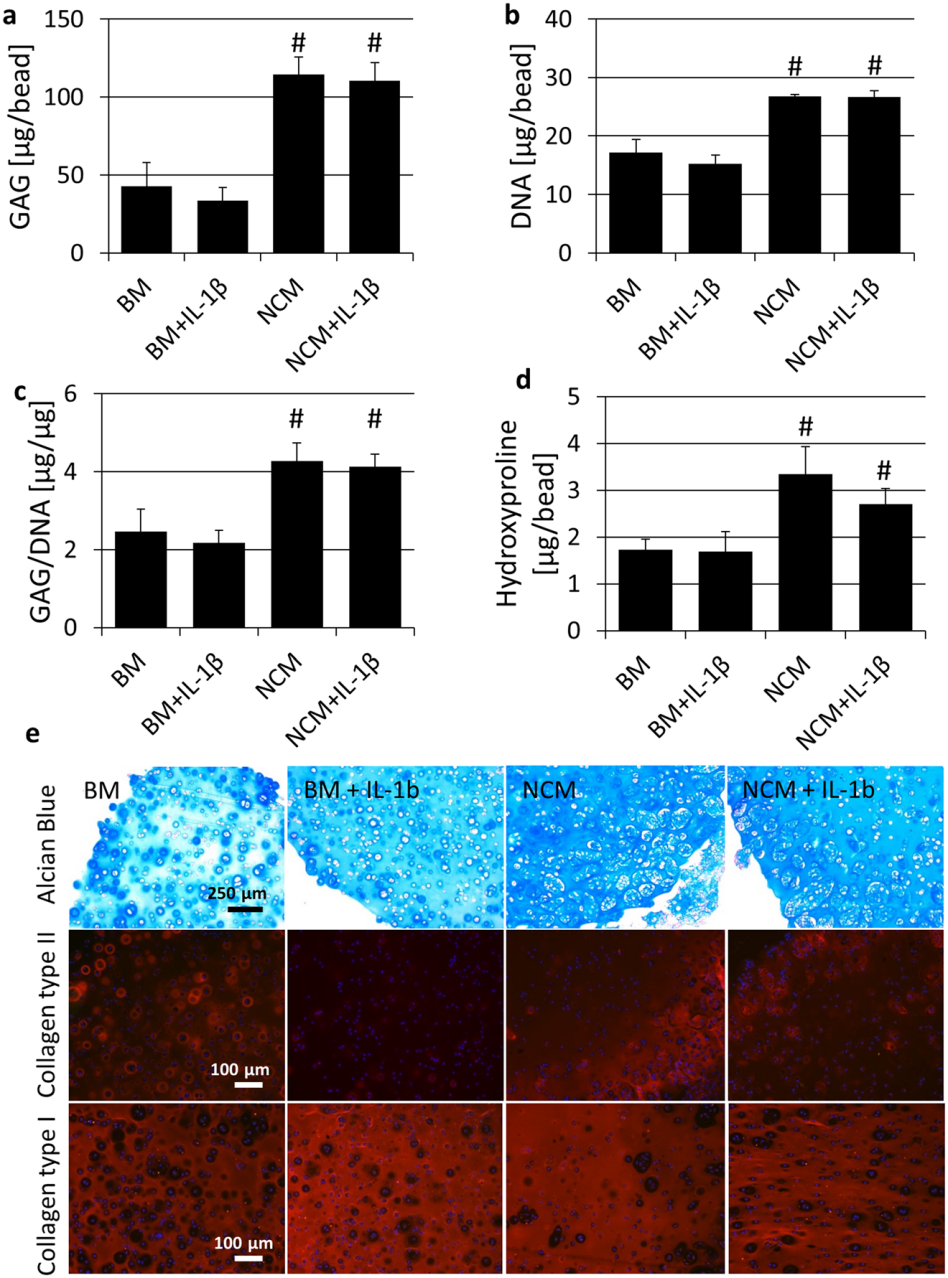
Addition of an inflammatory stimulus did not affect NCM’s regenerative potential (**a**) Glycosaminoglycan (GAG) and (**b**) DNA content per alginate bead seeded with bovine chondrocytes, (**c**) GAG per DNA and (**d**) hydroxyproline content per bead. Values represent means + standard deviations, n = 5 independent repeats per group. ^#^Indicates p < 0.05 from both base medium (BM) groups. (**e**) Alcian blue staining confirms increased GAG deposition with both NCM groups compared to both BM groups. Collagen type II staining was less intense with addition of IL-1β to BM relative to BM alone which, albeit to a lesser extent, is also observed with addition of IL-1β to NCM. Collagen type I deposition appeared to increase with addition of IL-1β to BM, though this is not observed with addition of IL-1β to NCM.

To determine whether NCM also has regenerative potential in the presence of an inflammatory stimulus, chondrocyte-seeded alginate beads were cultured in BM and NCM with and without addition of IL-1β. However, addition of IL-1β to BM and NCM did not affect GAG, DNA, GAG per DNA and hydroxyproline content compared to their counterparts without IL-1β (Fig. 3a–d). Alcian blue staining verified the increased GAG content with NCM with and without IL-1β compared to BM with and without IL-1β (Fig. 3e). Interestingly, immunostaining indicated that collagen type II is diminished with addition of IL-1β to BM, whereas this was not as clearly observed with addition of IL-1β to NCM. Furthermore, Addition of IL-1β appeared to increase the production of collagen type I in BM, but not in NCM.

**Figure 4:**
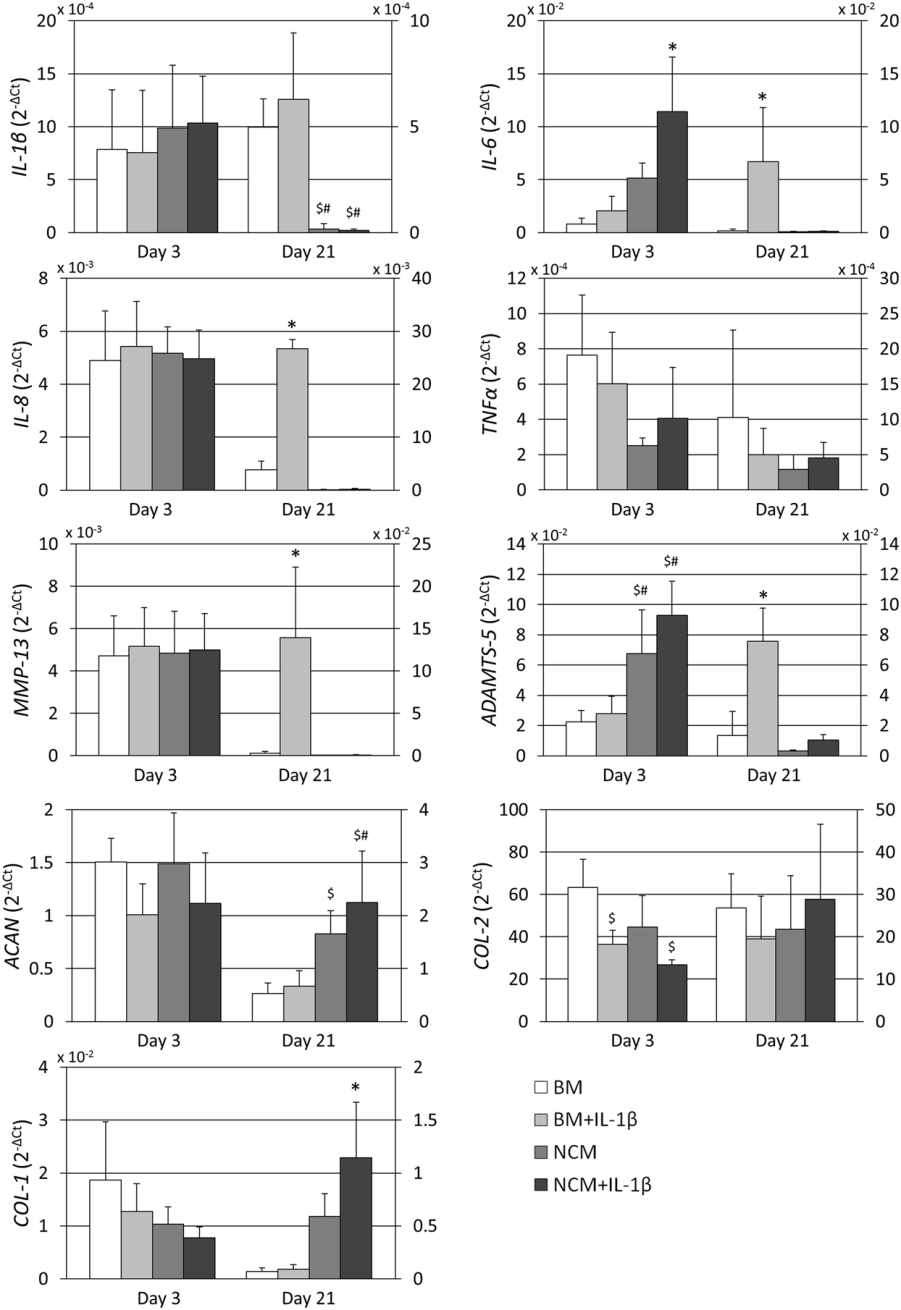
NC-rich nucleus pulposus matrix (NCM) may have anti-inflammatory and -catabolic potential. *IL*-*1β*/6/8: interleukin-1β/6/8; *TNFα*: tumor necrosis factor α; *ADAMTS*-5: a disintegrin and metalloproteinase with thrombospondin motifs 5; *MMP*-13: matrix metalloproteinase 13; *ACAN*: aggrecan; *COL*-2: collagen type II alpha 1. *COL*-1: collagen type I alpha 1. Expression levels are relative to 60 S ribosomal protein L13 (*RPL13*). Values are means + standard deviations, n = 4–5 independent repeats per group. *Indicates p < 0.05 compared to all other groups from the same time point, ^$^indicates p < 0.05 compared to base medium (BM), ^#^indicates p < 0.05 compared to BM + IL-1β.

No differences in expression of *IL*-*1β* were observed between culture groups at day 3, whereas at day 21 *IL*-*1β* expression was significantly lower in both NCM groups compared to both BM groups (Fig. 4). Furthermore, addition of IL-1β did not increase *IL*-*1β* expression in either BM or NCM relative to their non-treated control. Expression of *IL*-6 was significantly higher in NCM with IL-1β compared to all other groups at day 3. However, at day 21 its expression was significantly higher in BM with IL-1β compared to BM alone and both NCM groups. No differences in expression of *IL*-8 were observed at day 3, whereas its expression was significantly higher in BM with IL-1β compared to all other culture groups. No significant differences between culture groups were observed for *TNFα* at either day 3 or day 21. At day 3, no significant differences in expression of *MMP*-13 were observed, but its expression at day 21 was significantly higher in BM with IL-1β compared to BM alone and NCM groups. *ADAMTS*-5 expression at day 3 was significantly higher in NCM with and without IL-1β compared to both BM groups. However, at day 21 its expression was significantly higher in BM with IL-1β compared to the other groups. No differences in *ACAN* expression were observed at day 3. At day 21 however, *ACAN* expression in NCM was significantly higher compared to BM alone, and in NCM with IL-1β it was significantly higher compared to both BM groups. At day 3, addition of IL-1β significantly decreased *COL*-2 expression compared to BM alone, whereas no significant differences were observed at day 21. No differences were observed for *COL*-1 expression at day 3, but at day 21 its expression in NCM with IL-1β was significantly higher compared to all other culture groups.

### NCM lubrication

**Figure 5:**
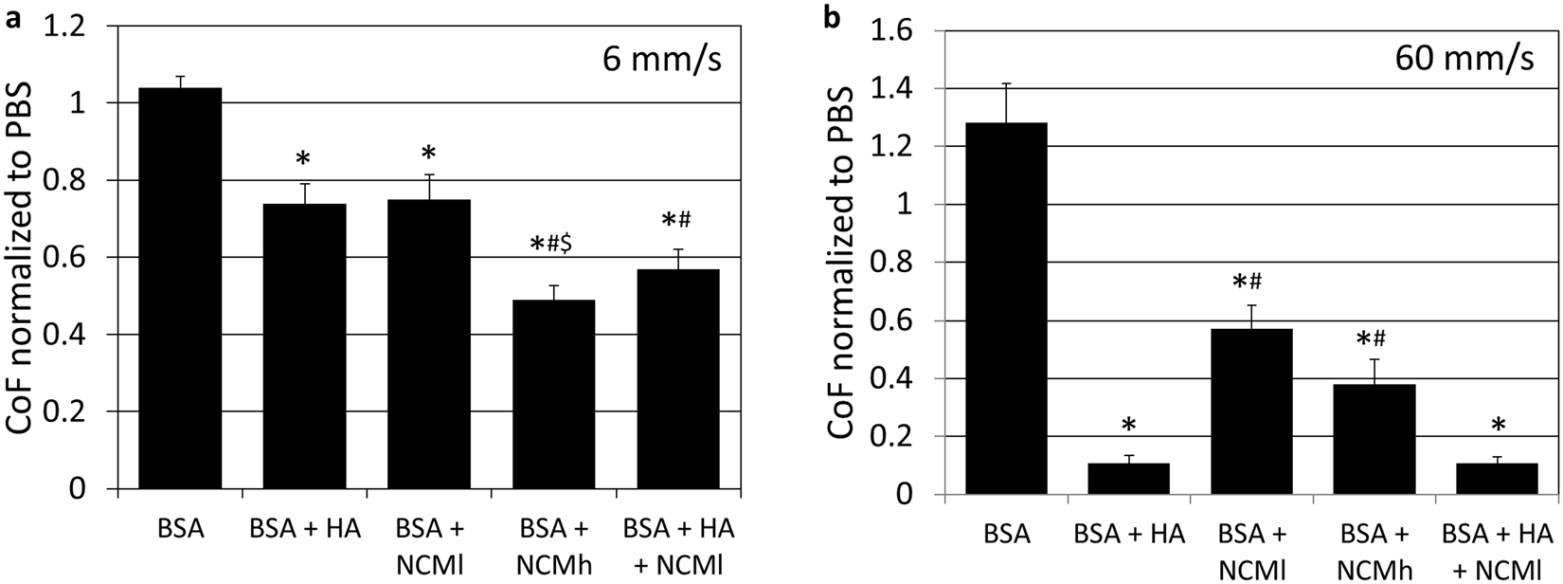
NC-rich nucleus pulposus matrix (NCM) has potential in cartilage lubrication. Coefficients of friction (COF) at cycle 20 in the different lubricants normalized to COF at cycle 20 in PBS alone at (**a**) 6 mm/s and (**b**) 60 mm/s. BSA: 5 mg/ml bovine serum albumin, HA: 4 mg/ml hyaluronic acid, NCMl: 4 mg/ml NCM, NCMh: 10 mg/ml NCM. Values are mean + standard error, n = 5 repeats for 6 mm/s measurements, n = 3 repeats for 60 mm/s measurements. *Indicates p < 0.05 compared to BSA, ^#^indicates p < 0.05 compared to BSA + HA, ^$^indicates p < 0.05 compared to BSA + NCMl.

At both 6 and 60 mm/s (Fig. 5a,b) addition of BSA to PBS either made no difference or caused slight increase in coefficient of friction (CoF). At 6 mm/s, addition of HA and lower amounts of NCM (NCMl) resulted in a significant decrease (by ~27%) in CoF after 20 cycles of sliding, compared to PBS with BSA (Fig. 5a). Combined addition of NCMl and HA resulted in a stronger reduction (45%) in CoF, which was significantly lower compared to BSA alone and BSA with HA, but not compared to BSA with NCMl. The strongest reduction (53%) in CoF was observed with addition of NCMh, where the CoF was significantly lower compared to BSA as well as both BSA with HA and BSA with NCMl.

**Figure 6:**
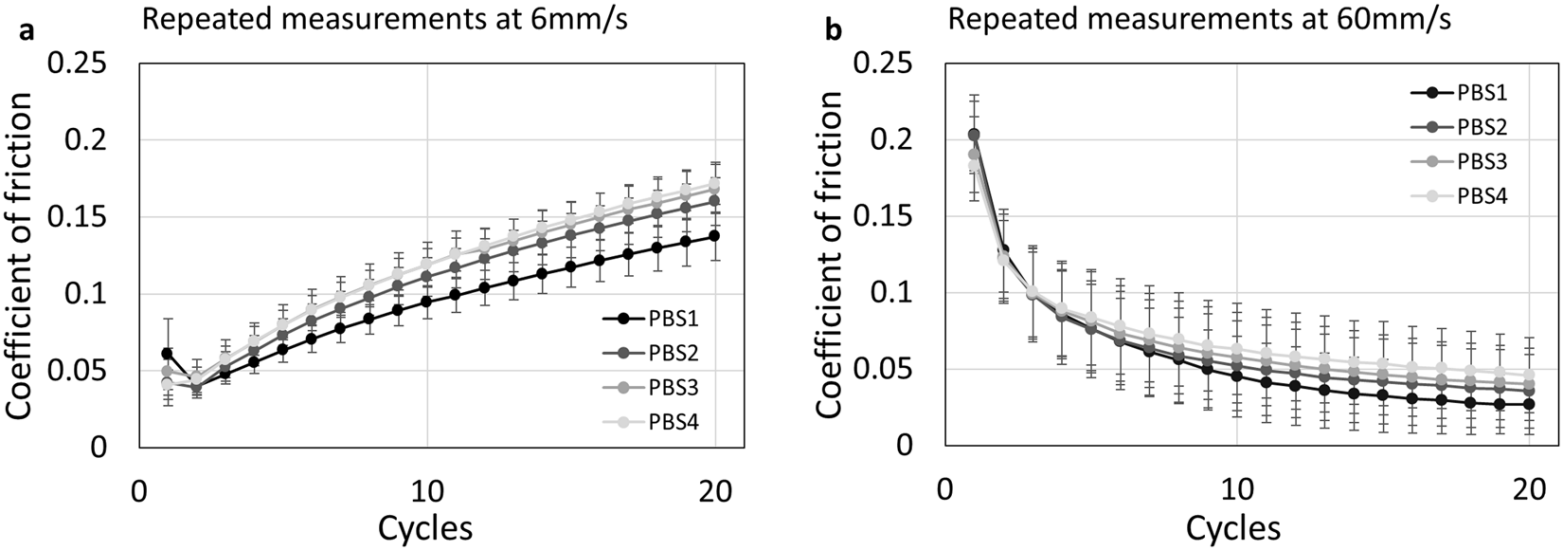
Repeated rounds of reciprocating sliding of cartilage against glass in PBS do not affect coefficients of friction (CoF). CoF for each cycle of 4 consecutive rounds (PBS1–PBS4, n = 4 repeats) of cartilage on glass sliding at (**a**) 6 mm/s and (**b**) 60 mm/s. Significant differences were observed between multiple rounds of sliding at either of the test speeds for individual measurements.

At 60 mm/s, addition of HA resulted in a 92% decrease in CoF after 20 cycles of sliding, compared to PBS with BSA, addition of HA and NCMl also showed a similar decrease. Addition of NCMl and NCMh respectively caused a significant decrease by 55 and 70% as compared to PBS with BSA (Fig. 5b). To verify that repeated sliding of the same plug did not affect CoF measurements, osteochondral plugs were slid against the glass surface for 4 rounds of 20 cycles, each round in fresh PBS. CoFs did not significantly change at either 6 (Fig. 6a) or 60 mm/s (Fig. 6b) as a result of multiple rounds of sliding. Therefore, no corrections were applied to the data presented in Fig. 5a and b.

## Discussion

NC-secreted factors, applied in the form of conditioned medium, have shown anabolic, proliferative and chondrogenic potential on NPCs and BMSCs^12–17^. Due to similarities between NPCs and chondrocytes, a translation of NC-secreted factors from IVD applications to the field of chondrocytes/cartilage seems logical. Indeed, NC-conditioned medium recently also demonstrated anabolic and anti-inflammatory effects on human OA chondrocytes. Since direct application of NCM had even stronger anabolic effects on NPCs compared to NC-conditioned medium^18^, it seemed plausible that NCM would have stimulatory effects on chondrocytes as well. Hence, this study tested the feasibility of a novel NCM approach, as a bioactive viscosupplementation lubricant, to minimize joint pain upon injection in the OA joint, while simultaneously providing a regenerative stimulus to the resident chondrocytes.

Previous findings regarding NC-conditioned medium and NCM are in line with the current results, since NCM exerted strong anabolic effects on bovine chondrocytes as shown by increased GAG, DNA GAG per DNA and hydroxyproline content with NCM compared to BM. NCM resulted in even higher GAG and DNA per bead content compared to addition of 10 ng/ml TGF-β1. Because the number of chondrocytes decreases with OA, increased cell proliferation thus seems beneficial. However, it remains to be determined whether this proliferative effect persists when NCM is applied on cartilage tissue, and whether the OA joint *in vivo* may be able to support such increase in cellularity. Collagen immunohistochemistry revealed increased deposition of collagen type I with NCM compared to BM and TGF, which is in line with the increased expression of *COL*-1 with NCM at day 21. Differences in collagen type II deposition between culture groups were less discernible, although collagen type II was consistently present mainly at the edges of the beads with NCM, whereas it was deposited throughout the bead with addition of TGF-β1. This may indicate that TGF-β1 is smaller than the active component(s) of NCM and can diffuse into the bead more easily. As such, it would exert its effects on a higher number of cells, which would explain the higher *ACAN* and *COL*-2 gene expression levels observed with TGF-β1 compared to NCM. In line with this, in a previous study where NCM’s regenerative potential was tested on NPC-seeded alginate beads, NCM appeared to enhance collagen type II deposition throughout the alginate beads, rather than collagen type I^18^. In that study, NPCs were seeded at a density of 3 million cells/ml, whereas in this study chondrocytes were seeded at 10 million cells/ml, indicating that limited diffusion of NCM’s bioactive factors may play a larger role in the current study. Alternatively, it is possible that NPCs and chondrocytes, despite their similarities, respond differently to NCM stimulation in terms of collagen deposition, or that the NPCs used in the previous study where in a healthier state compared to the chondrocytes in the current study. Nonetheless, despite NCM’s stimulation of collagen type I rather than type II, it exhibits strong matrix anabolic effects, and further studies are required for a more in-depth characterization of these effects under disease conditions.

Inflammatory cytokines, which in turn stimulate the release of catabolic factors such as MMPs and ADAMTSs, play a central role in the onset and progression of OA. Therefore, this study tested whether NCM can also elicit a regenerative response of chondrocytes in the presence of an inflammatory stimulus. Gene expression results, mainly at day 21, suggest that addition of IL-1β to BM indeed resulted in an inflammatory environment, as shown by increased *IL*-6 and *IL*-8 expression levels. Moreover, IL-1β induced catabolism on the gene level as shown by increased *MMP*-13 and *ADAMTS*-5 expression levels at day 21 in BM. However, treatment of the NCM group with IL-1β did not result in increased expression levels of these genes relative to BM or NCM alone, suggesting that NCM has anti-inflammatory and –catabolic potential, as also observed in previous *in vitro* and *in vivo* studies testing the effects of NCM on NPCs^18,19^. Interestingly *IL*-*1β* and *TNFα* did not respond to addition of IL-1β to either BM or NCM, which is not in line with previous findings^22^. However, a previous study reported that IL-1β induced increased *IL*-*1β* gene expression by nucleus pulposus cells from degenerate tissues but not by nucleus pulposus cells from healthy tissues^23^. The bovine donors in the current study were relatively young, and since IVD degeneration is associated with aging, the cells may have been less sensitive to IL-1β treatment. Nonetheless, *IL*-*1β* gene expression at day 21 was significantly lower in both NCM groups relative to both BM groups, which underscores NCM’s anti-inflammatory potential.

The inflammatory stimulus applied during culture did not significantly affect GAG, DNA and hydroxyproline content, indicating that NCM’s anabolic and proliferative effects are maintained in an inflammatory environment. This is also verified by *ACAN* gene expression levels, which were not significantly different in NCM treated with IL-1β compared to NCM alone. However, NCM in combination with IL-1β seemed to further induce unfavorable collagen production, as observed by decreased collagen type II staining intensity and *COL*-2 gene expression at day 3, and increased *COL*-1 gene expression at day 21 for NCM treated with IL-1β. At day 21 however, no significant differences in *COL*-2 expression levels were observed, which suggests that collagen type II production has recovered over the culture time, despite the continuous presence of IL-1β. Furthermore, a supra-physiological concentration of IL-1β was applied, and NCM’s potential in an inflammatory environment should be further investigated in a more physiological setting, e.g. *in vivo*.

In addition to its regenerative effects on chondrocytes, this study shows that low concentrations (4 and 10 mg/ml) of NCM solutions were capable of reducing cartilage CoF. Thus NCM may be applied as an OA joint lubricant with or without HA. Tribological control measurements (i.e. 4 repeated rounds of reciprocating sliding of the same osteochondral plug, each time in fresh PBS) demonstrated that there were no significant differences between runs, and thus no corrections were applied to the other experiments. This strategy of using the same plug repeatedly is thus successful in avoiding differences in contact area due to joint to joint biological variations in cartilage properties.

At both 6 and 60 mm/s, addition of BSA to PBS either made no difference or caused slight increase in CoF. This was not unexpected. BSA is a non-glycosylated globular protein and was not expected to either provide boundary or hydrodynamic lubrication. Moreover, albumin has been implicated in interfering with lubricin (PRG4) adsorption on natural cartilage^24^ and biomaterial^25^ surfaces. BSA is an abundant synovial fluid protein thus it’s effect was necessary to monitor in our measurements (all lubricant solutions contained 5 mg/ml BSA except PBS controls).

At low speeds, e.g. 6 mm/s, neither hydrodynamic^6,7^ nor tribohydration^26^ mechanisms of cartilage lubrication are expected to apply. At this speed in natural cartilage, adsorbed molecules on the cartilage surface provide boundary lubrication. Lubricin (PRG4) is an important glycoprotein bound to the articulating surface of AC, i.e. the lamina splendens. It is anchored in a looped or one-end-free fashion providing boundary lubrication. In addition to PRG4, surface active phospholipids (SAPL) belong to the most researched boundary lubricants^27–29^. As the NP tissue has no lubricating function, NCM, which is simply composed of NP matrix components, was not expected to act as a boundary lubricant. In a previous study investigating the proteomic contents of porcine NC-conditioned medium, PRG4 was not shown to be present^30^. Also notochordal cell vacuoles are speculated to contain lipids^31^, but their surface-activity has never reported on. Finally, the mucopolysaccharides present in the NC matrix may also be surface active and give rise to boundary lubrication. However, past studies reported that concentrations as high as 100 mg/ml of chondroitin sulfate was necessary to decrease the coefficient of friction^32^ whereas in this study the concentration of NCM used would have resulted in only 1.5 and 4 mg/ml of GAGs. Thus, it was surprising that boundary lubrication was NCM’s stronger mode of action, i.e. more than at higher speeds, and some component other than PRG4 would be providing boundary lubrication to the cartilage at these lower speeds. The fact that addition of NCM induced a dose-dependent decrease in CoF would be consistent with its boundary lubricant mechanism. At 4 mg/ml, this effect was similar to HA at a concentration similar to that used clinically for viscosupplementation. Finally, when added to HA, it further decreased the CoF indicating absence of any antagonistic interaction between HA and NCM and perhaps a differing mode of action.

At higher speeds e.g. 60 mm/s, hydrodynamic lubrication, where a wedge of fluid is created when opposing cartilage surfaces slide on each other^6,7,33^, comes into play, and furthermore, tribohydration^26^ will become active. This type of lubrication depends, among others, on the viscosity of the trapped fluid. As NCM is rich in mucopolysaccharides^30^, it might be expected to have some lubricating effects in this fashion. However, during the experiments, it was visually observed that a 4 mg/ml HA solution was more viscous compared to NCMl (4 mg/ml) and even NCMh (10 mg/ml) solutions, and it was doubtful that NCM would be effective. Nevertheless, NCM had a lubricating effect at this test speed, as it reduced the CoF ~55% at 4 mg/ml and ~70% at 10 mg/ml relative to PBS + BSA, even though this was less of a decrease in CoF then that with HA. This could suggest that the lubricating mechanism of NCM at higher speeds may not purely be through its viscosity but also due to other unknown effects. Again, at this speed, the combination of NCMl with HA induced a similar reduction in CoF to HA alone, indicating no antagonism and that this combination may ultimately be applied clinically in order to maximize the lubricating properties while still benefitting from NCM’s regenerative potential.

Before clinical application can be considered, further processing of porcine NCM is required. α-Gal is an antigen present on the surface of porcine cells and is a major cause of rejection in pig-to-human xenotransplantation^34^. α-Gal should therefore be removed from porcine NCM prior to *in vivo* applications, which could be achieved through enzymatic treatment with α-Galactosidase^35^. Furthermore, porcine genetic material contains endogenous retroviruses (PERVs) that can be expressed upon implantation in other species^36^. As such, porcine NCM requires processing to remove this genetic material as well.

In conclusion, this study demonstrates that NCM exerts regenerative effects on bovine chondrocytes and has strong lubricating properties on articular cartilage. Therefore, NCM holds promise as a therapy for OA, where it may be applied to minimize pain directly upon injection into the joint, while simultaneously inducing a regenerative stimulus to the resident chondrocytes, that may restore the affected cartilage tissue towards a healthy state. Further studies should focus on NCM’s regenerative effects in a more physiological model, and on processing methods for the clinical application of NCM.

## Materials and Methods

### Production of porcine NCM

NC-rich NP tissue was harvested from the IVDs of porcine donors (n = 5, ~3 months old). The tissue was lyophilized (Labconco, Kansas City, MO, USA) overnight, resulting in a dry and brittle matrix, which was subsequently pulverized using a microdismembrator (Sartorius, Goetingen, Germany). The NCM powder was aliquoted and stored at −80 °C until further use.

### Chondrocyte isolation and alginate bead production

Full-depth slices of articular cartilage where collected from the metacarpal-phalangeal joints of bovine donors (n = 5, ~3 years old), and collected in phosphate-buffered saline (PBS) with 15% Penicillin-Streptomycin (P/S). Subsequently, cartilage flakes where incubated for 20 min at 37 °C and 5% CO_2_ in the presence of 0.1% Amphotericin B. Thereafter, the PBS-P/S-Amphotericin mixture was aspirated and cartilage flakes were digested overnight in digestion medium (hgDMEM supplemented with 10% fetal bovine serum (FBS), 1% P/S, 0.1% Amphotericin B and 0.5% collagenase type II) at 37 °C and 5% CO_2_. The following day, the cells suspension was strained using a 70 µm cell strainer and chondrocytes were washed twice in fresh hgDMEM. Chondrocytes were resuspended in 1.2% alginate (Sigma, 180947, Zwijndrecht, the Netherlands) at 10 million cells/ml, and alginate beads were produced according to a previous protocol (Guo *et al*., 1989). Briefly, 10 million cells were mixed with 1 ml alginate using an 18 G mixing needle after which the suspension was aspirated in a syringe. Alginate beads were produced by dropping the cell suspension in a 102 mM calcium chloride (Merck, 102378) solution. Subsequently, beads where washed 3 times with 0.9% sodium chloride (Merck, 106404) solution before being transferred to culture medium.

### Alginate bead culture

To test NCM’s anabolic effect, chondrocyte-seeded alginate beads were cultured in base medium (BM: hgDMEM supplemented with 1% P/S, 1% ITS-1+ (Corning, 354352, Lasne, Belgium), 50 mg/ml ascorbic acid-2-phosphate (Sigma, A8960), 1.25 mg/ml bovine serum albumin (Roche, 10735078001) and 40 mg/ml L-proline (Sigma, P5607)), NCM (2 mg/ml NCM added to BM) or with addition of 10 ng/ml TGF-β1 as a positive control. Furthermore, to test whether NCM has a regenerative effect in an inflammatory environment, alginate beads were also cultured in BM and NCM in the presence of 5 ng/ml IL-1β. Alginate beads were cultured for 3 weeks at 37 °C and 5% CO_2_ with medium changes twice per week. NCM, TGF-β1 and IL-1β were added with each medium change. After culture, alginate beads were stored at −80 °C for biochemical assays (day 0 and 21) or gene expression analysis (day 3 and 21), or embedded in paraffin for (immuno)histochemical staining (day 28).

### Biochemical content and (immuno)histochemical staining

To determine the biochemical content, alginate beads were digested overnight at 60 °C in papain digestion buffer (100 mM phosphate buffer (Sigma, P5244), 5 mM l-cysteine (Sigma, 200-157-7), 5 mM ethylene diamine tetra-acetic acid (Sigma, 03620), and 140 mg/mL papain (Sigma, P4762). From the digested samples, GAG content was determined with dimethyl-methylene blue (DMMB) assay, modified from a previous protocol^37^ where shark cartilage chondroitin sulfate (Sigma, C4384) was used as a reference. Hydroxyproline content was measured using the Chloramin-T assay^38^ with a trans-4-hydroxyproline (Sigma, H54409) reference. DNA content was measured using the Qubit Quantification Platform (Invitrogen).

Paraffin-embedded alginate beads were sectioned and stained with Alcian blue and hematoxylin for visualization of proteoglycan deposition and cell nuclei. For collagen immuno-staining, sections were first dewaxed using xylene and a series of decreasing ethanol concentration. Sections were washed in PBS for 5 minutes and antigen retrieval was performed with citrate buffer for 20 minutes at 96 °C for collagen type I staining, and with 0.05% pepsine in 10 mM HCl for 5 minutes at 37 °C for collagen type II. Samples were washed again twice with PBS with 0.1% tween, and subsequently blocked with 10% normal goat serum for 30 minutes. Samples were then incubated overnight at 4 °C with the primary antibody in 1% NGS in PBS (Abcam, Ab34710, 1:200 dilution for collagen type I and Abcam, Ab180697, 1:200 dilution for collagen type II). The next day, samples were washed twice for 5 minutes in PBS with 0.1% tween, followed by incubation with the secondary antibody (Molecular Probes anti-rabbit IgG, A21428, 1:200 dilution for collagen type I and Molecular Probes anti-mouse IgG2a, A21137, 1:300 dilution for collagen type II) and DAPI (1:500 in PBS). Thereafter, samples were washed again twice in 0.1% tween in PBS and embedded using mowiol. Pictures were taken using a fluorescent microscope (Zeiss Axiovert 200 M, Zeiss, Sliedrecht, the Netherlands). Positive control samples (bovine tendon for collagen type I and articular cartilage for collagen type II) were included, as well as negative controls for each sample (i.e. omission of the primary antibody). The negative controls showed no aspecific positive staining.

### Gene expression

Gene expression analysis was performed on 3 alginate beads pooled per group. Alginate beads were dissolved in sodium citrate buffer (55 mM trisodiumcitrate-2-hydrate (Merck, 1064480500), 0.15 M sodium chloride, 25 mM HEPES (Sigma, H3375) in RNAse-free water, pH adjusted to 7.4) for 5 minutes at room temperature. After centrifugation the cell pellet was lysed in 300 µl RLT buffer (Qiagen, 74104, Venlo, The Netherlands) with 1% β-mercapto-ethanol. RNA was extracted and purified using the Qiagen mini-kit (Qiagen, 74104) with an on-column DNAse digestion step. A spectrophotometer (ND-1000, Isogen, de Meern, The Netherlands) was used to test the quantity and purity of isolated RNA. The absence of genomic DNA was verified with a minus-RT reaction (iCycler; Bio-Rad, Veenendaal, The Netherlands). cDNA was synthesized using the VILO-kit (Invitrogen, 11754050). The tested genes and their corresponding primer pairs are listed in Table 1. Ribosomal protein L-13 (RPL-13) was selected as the reference gene as its expression was most stable throughout all culture conditions. Gene expression was investigated using real-time qPCR (CFX384, Bio-Rad) and expression is reported according to the 2^−ΔCt^ method. In order to be able to make statistical comparisons, Ct values were set to 40 for samples from which no signal was obtained.

**Table 1:** Primer sequences for target and reference genes used in RT-qPCR assays.

**Gene**	**Accession number**	**Oligonucleotide sequence** (5′ → 3′)	**Product size (bp)**
*RPL13*	NM_001076998	FW: CTGCCCCACAAGACCAAG RV: TTGCGAGTAGGCTTCAGAC	140
*IL-1β*	NM_174093	FW: AGCATCCTTTCATTCATCTTTGAAG RV: GGGTGCGTCACACAGAAACTC	88
*IL-*6	NM_173923	FW: GGGCTCCCATGATTGTGGTA RV: GTGTGCCCAGTGGACAGGTT	69
*IL-*8	NM_173925.2	FW: TGCTTTTTTGTTTTCGGTTTTTG RV: AACAGGCACTCGGGAATCCT	71
*TNFα*	NM_173966	FW: ACACCATGAGCACCAAAAGC RV: GCAACCAGGAGGAAGGAGAA	130
*ADAMTS-*5	NM_001166515	FW: TCACTGCCTACTTAGCCCTGAA RV: GCTCCAACCGCTGTAGTTCAT	125
*MMP-*13	NM_174389	FW: CTTGTTGCTGCCCATGAGTT RV: TTGTCTGGCGTTTTGGGATG	197
*ACAN*	NM_173981	FW: CCAACGAAACCTATGACGTGTACT RV: GCACTCGTTGGCTGCCTC	107
*COL2A1*	NM_001113224	FW: TGGCTGACCTGACCTGAC RV: GGGCGTTTGACTCACTCC	187
*COL1A1*	NM_174520	FW: TGAGAGAGGGGTTGTTGGAC RV: AGGTTCACCCTTCACACCTG	142

*RPL13*: 60S Ribosomal Protein L13; *IL*-*1β*/*6*/*8*: interleukin-1β/6/8; *TNFα*: tumor necrosis factor α; *ADAMTS*-*5*: a disintegrin and metalloproteinase with thrombospondin motifs 5; *MMP*-*13*: matrix metalloproteinase 13; *ACAN*: aggrecan; *COL2A1*: collagen type II alpha 1. The annealing temperature of all primer pairs was 60 °C.

### Preparation of bovine osteochondral plugs

Bovine stifle joints (n = 4, 2 years, bulls) where acquired from Kroon Vlees b.v., Groningen, The Netherlands. Excessive skin was removed and the joined was opened, careful not to damage the cartilage surface. A total of 14 osteochondral plugs with a diameter of 12 mm where drilled from the femoral condyles using a hollow drill bit. During drilling, the cartilage was continuously wetted with PBS to prevent overheating of the samples. After removal of the osteochondral plugs from the joint, they were kept in PBS on ice until they were used for tribological tests, within 2 hours.

### Tribological testing

Tribological tests were performed in reciprocating sliding using UMT-3 (Universal Mechanical Tester, Bruker Corporation, USA). A glass surface was fixed in a warmed basin (33 °C, the temperature of the knee joint) to allow a film of liquids/lubricants to cover the surface. Osteochondral plugs were mounted on a load cell and slid against the glass surface in a reciprocating configuration. For these tests, the osteochondral plugs were divided in three groups. For group 1 (n = 5), the glass surface was first submerged in PBS after which the plug was pressed against the glass until a normal load of 4N was measured, which will result in a low contact pressure of <0.25 MPa as used in recent studies^26^. Then reciprocating sliding was performed for 20 cycles (55 mm one-way) at 6 mm/s under a constant normal load, directly followed by 20 cycles at 60 mm/s. Thereafter the plug was unloaded and PBS was aspirated and replaced by PBS with 5 mg/ml bovine serum albumin (PBS + BSA) and the test was repeated. Thereafter, this procedure was also repeated with PBS + BSA with 4 mg/ml hyaluronic acid (PBS + BSA + HA) and with PBS + BSA + HA with 4 mg/ml NCM (PBS + BSA + HA + NCM). For group 2 (n = 5) the same test was performed with different medium groups: PBS, PBS + BSA, PBS + BSA with 4 mg/ml NCM (PBS + BSA + NCMl), and PBS + BSA with 10 mg/ml NCM (PBS + BSA + NCMh). During the tests, normal forces and friction forces were monitored, and a custom Matlab script was used to filter the data to incorporate only the data points where the test speed was close to the set speed (a 10% error was allowed), and to calculate the CoF. CoFs obtained at the 20^th^ cycle for each measurement were normalized to their respective measurement in PBS alone, to correct for contact area differences. For group 3 (n = 4) osteochondral plugs, the same procedure was performed, but each round in fresh PBS to verify that repeated sliding at two speeds did not affect the cartilage surface and thus the measured CoF values.

### Statistics

Statistics were performed with Statistical Package for Social Sciences (SPSS, version 22; IBM, Armonk, NY). Normality was tested using the Shapiro-Wilk test. For biochemical and gene expression data, one-way analysis of variance (ANOVA) was performed, followed by independent t-tests post hoc testing with Bonferroni corrections. For gene expression data, a one-way, rather than a two-way ANOVA was performed at each time point, since only differences between medium groups were of interest and not the factor time. For tribological data, normalized CoF values from different lubricant groups were compared with paired (for intra-plug comparisons) or unpaired t-tests (for inter-plug comparisons), in post-hoc fashion with Bonferroni corrections. For PBS control measurements repeated measures ANOVAs were used to compare the CoF values from 4 sequential tests consisting each of 20 cycles at 6 and 60 mm/s.

### Data availability statement

The datasets generated during and/or analyzed during the current study are available from the corresponding author on reasonable request.
